# The Duffy binding protein (PkDBPαII) of *Plasmodium knowlesi* from Peninsular Malaysia and Malaysian Borneo show different binding activity level to human erythrocytes

**DOI:** 10.1186/s12936-017-1984-8

**Published:** 2017-08-11

**Authors:** Khai Lone Lim, Amirah Amir, Yee Ling Lau, Mun Yik Fong

**Affiliations:** Department of Parasitology, Faculty of Medicine, University of Malaya, 50603 Kuala Lumpur, Malaysia

**Keywords:** *Plasmodium knowlesi*, Malaysia, Duffy binding protein, DARC, Erythrocyte binding

## Abstract

**Background:**

The zoonotic *Plasmodium knowlesi* is a major cause of human malaria in Malaysia. This parasite uses the Duffy binding protein (PkDBPαII) to interact with the Duffy antigen receptor for chemokines (DARC) receptor on human and macaque erythrocytes to initiate invasion. Previous studies on *P. knowlesi* have reported distinct Peninsular Malaysia and Malaysian Borneo PkDBPαII haplotypes. In the present study, the differential binding activity of these haplotypes with human and macaque (*Macaca fascicularis*) erythrocytes was investigated.

**Methods:**

The PkDBPαII of Peninsular Malaysia and Malaysian Borneo were expressed on the surface of COS-7 cells and tested with human and monkey erythrocytes, with and without anti-Fy6 (anti-Duffy) monoclonal antibody treatment. Binding activity level was determined by counting the number of rosettes formed between the transfected COS-7 cells and the erythrocytes.

**Results:**

Anti-Fy6 treatment was shown to completely block the binding of human erythrocytes with the transfected COS-7 cells, thus verifying the specific binding of human DARC with PkDBPαII. Interestingly, the PkDBPαII of Peninsular Malaysia displayed a higher binding activity with human erythrocytes when compared with the Malaysian Borneo PkDBPαII haplotype (mean number of rosettes formed = 156.89 ± 6.62 and 46.00 ± 3.57, respectively; *P* < 0.0001). However, no difference in binding activity level was seen in the binding assay using *M. fascicularis* erythrocytes.

**Conclusion:**

This study is the first report of phenotypic difference between PkDBPαII haplotypes. The biological implication of this finding is yet to be determined. Therefore, further studies need to be carried out to determine whether this differential binding level can be associated with severity of knowlesi malaria in human.

## Background

Malaria is a parasitic disease that affects millions of people in Africa, Asia, and South and Central America. In 2015, there were approximately 212 million new cases of malaria, causing nearly 429,000 deaths [1]. This mosquito-borne disease is caused by blood protozoa of the genus *Plasmodium.* Four species, *Plasmodium falciparum*, *Plasmodium vivax*, *Plasmodium malariae*, and *Plasmodium ovale* (now comprises of *P. ovale wallikeri* and *P. ovale curtisi*) have long been known to cause human malaria [2, 3]. Over the past decade, the simian malaria parasite *Plasmodium knowlesi* has emerged to cause significant human infections in Southeast Asia [4–8]. Microscopically, *P. knowlesi* bears a resemblance to the benign *P. malariae*, but it has a unique 24-h erythrocytic cycle (quotidian fever pattern), and it can cause severe disease and death if left untreated. Knowlesi malaria is now the most common form of malaria in Malaysia especially in Malaysian Borneo [9–11].

*Plasmodium* merozoites invasion into erythrocyte is a complex process which involves attachment, apical reorientation, tight-junction formation and entry into a parasitophorous vacuole. These steps are mediated by specific molecular interactions between the parasite’s ligand and its corresponding receptor on the surface of erythrocyte membrane [12]. Both *P. knowlesi* and *P. vivax* are known to interact with the Duffy antigen receptor for chemokines (DARC) to invade Duffy-positive human erythrocytes. Duffy-negative human erythrocytes are refractory to invasion by these two *Plasmodium* species [13–15]. The invasion of human erythrocytes by *P. knowlesi* is dependent on the interaction of DARC with the parasite’s ligand, the Duffy binding protein (PkDBPα) [16]. PkDBPα can be divided into seven regions (I–VII). The N-terminal cysteine rich region II (PkDBPαII) contains the critical Duffy-binding-like (DBL) ligand domain for binding to the erythrocyte [17, 18]. PkDBPαII has been shown to bind to Duffy-positive human and macaque erythrocytes [19]. On the other hand, two other related proteins, the PkβII and PkγII, bind only to macaque erythrocytes [17].

The clinical symptoms of malaria are primarily attributed to the blood-stage of the parasite life cycle, which results from repeated rounds of erythrocyte invasion, parasite multiplication, erythrocyte lysis and release of free merozoites. It has been observed that antibodies raised against PkDBPαII could inhibit *P. knowlesi* invasion of human and macaque erythrocytes in vitro [20], making it a possible target vaccine candidate against knowlesi malaria.

Although knowlesi malaria is seen in both Peninsular Malaysia and Malaysian Borneo, hyperparasitaemia and severe cases are more prominent in Malaysian Borneo [10, 21–27]. Previous studies have shown genetic diversity in the PkDBPαII of clinical isolates from Peninsular Malaysia and Malaysian Borneo [28, 29]. It was further observed that PkDBPαII haplotypes from Peninsular Malaysia and Malaysian Borneo were genetically distinct. This led us to investigate whether the genotypic differences in PkDBPαII could influence the ability of the parasite for invasion into erythrocytes. In the present study, binding activity of Peninsular Malaysia and Malaysian Borneo PkDBPαII haplotypes with human and macaque (*Macaca fascicularis*) erythrocytes was compared using the erythrocyte-binding assay.

## Methods

### Blood samples and DNA preparation

Earlier studies on *P. knowlesi* clinical isolates reported distinct PkDBPαII haplotype groups from Peninsular Malaysia and Malaysian Borneo. The predominant haplotype in Peninsular Malaysia was haplotype H2, and H47 in Malaysian Borneo [28, 29]. For this study, haplotype H2 and H47 were represented by clinical isolates HAN and SBH31 respectively. The blood samples containing these two isolates were obtained from the earlier studies [28, 29]. For each isolate, the DNA was extracted from 100 µl of blood using QIAGEN blood DNA extraction kit (QIAGEN, Hilden, Germany).

For the erythrocyte-binding assay, erythrocytes were collected from fresh whole blood into lithium heparin tube. The Duffy genotype of the erythrocytes was determined via PCR method described previously [30]. The erythrocytes were washed using incomplete RPMI medium for at least three times and stored at 4 °C for a maximum of 7 days.

### Gene amplification and sequencing of PkDBPαII

The PkDBPαII region was amplified by PCR using primers containing a *Bgl*II restriction enzyme cut site, PkDBPαII-F1: 5′-GGCAGATCTGTTATTAATCAAACTTTTCTTC-3′ and PkDBPαII-R1: 5′-AGATCTGTTCAGTTATCGGATTAGAACTG-3′. The amplification reaction was performed using the following thermal cycling condition: 95 °C for 5 min, 30 cycles at 95 °C for 30 s, 55 °C for 45 s and 72 °C for 70 s, followed by a 10-min extension at 72 °C. GoTaq^®^ Flexi DNA Polymerase (Promega, Corp, USA) was used in the PCR. The PCR product of 1027 bp was purified using QIAquick PCR purification kit (QIAGEN, Hilden, Germany) following the manufacturer’s instructions. The purified PCR product was then ligated into cloning vector pGEM-T^®^ (Promega Corp, USA). Each ligation mixture was transformed into One-Shot^®^ TOP10 chemically competent *E. coli* cells (Invitrogen, Carlsbad, CA). Plasmid DNA of recombinant clones harbouring the PkDBPαII fragment was sent to a commercial laboratory (First BASE Laboratories Sdn Bhd, Malaysia) for DNA sequencing. Sequence analysis was performed on two clones for each parasite isolate.

### Construction of recombinant plasmids for surface expression on COS-7 cells

The plasmid pDisplay™ (Invitrogen, Carlsbad, CA) is an expression vector designed to target recombinant protein to the surface of mammalian cells. In this study, the fluorescent reporter gene *AcGFP* (green fluorescent protein from *Aequorea coerulescens*) was added to the C-terminal of the insert site of pDisplay™ to facilitate direct visualization of the expressed protein. The AcGFP gene was PCR-amplified from the plasmid pAcGFP1-C1 using the forward primer 5′-GTCGACGCCACCATGGTGAGCAAG-3, and reverse primer 5′-GTCGACCTTGTACAGCTCATCCATGCC-3′, which contained a *Sal*I restriction enzyme cut site. The AcGFP gene was then cloned and sequenced. Recombinant pGEM-T^®^ plasmid carrying the PkDBPαII and AcGFP genes was cleaved with *Bgl*II and *Sal*I, respectively, and then cloned into the corresponding sites in the pDisplay™ vector. The plasmid construct, designated as pDisplayAcGFP-PkDBPαII, was purified using a QIAprep Spin Miniprep Kit purification kit (QIAGEN, Hilden, Germany).

### Mammalian COS-7 cell transfection

COS-7 (ATCC^®^ CRL-1651™) cells were grown in DMEM-high glucose supplemented with 10% heat inactivated fetal bovine serum, 1 mM sodium pyruvate, 2 mM l-glutamine and 1% penicillin–streptomycin at 37 °C in a 5% CO_2_ incubator. All reagents used were from Gibco™ (Invitrogen, Carlsbad, CA). For transfection, COS-7 cells were plated into six-well culture plates and then transfected with the pDisplayAcGFP-PkDBPαII plasmid DNA (1 µg per well) using 10 µl Lipofectamine 3000 reagent (Invitrogen, Carlsbad, CA) in serum-free incomplete DMEM and grown at 37 °C in 5% CO_2_. After 24 h, the transfection medium was replaced with complete DMEM-high glucose, and the cells were incubated for another 24 h. The transfected COS-7 cells were used in the erythrocyte-binding assay.

### Erythrocyte-binding assay

Confluent monolayer of COS-7 cells was used 48 h after transfection. The transfected cells were incubated for 2 h at 37 °C with human erythrocytes [Duffy-positive Fy(a+b−) phenotype, blood group O^+^, 1% haematocrit in incomplete DMEM]. The cells were washed three times with PBS to remove non-adherent erythrocytes, and then incubated with 1% paraformaldehyde for 10 min at room temperature to stabilize the rosettes. The nuclei of COS-7 cells were stained with 1 μg/ml Hoechst 33,342 dye (Invitrogen, Carlsbad, CA) for 1 min. To determine transfection efficiency, unfixed green fluorescence cells were observed (200× magnification) and counted using fluorescence microscopy with a FITC-filter (488 nm excitation wavelength) mounted on a Nikon ECLIPSE TE300 inverted microscope. Positive rosettes were defined as adherent erythrocytes covering more than 50% of the COS-7 cell surface [31–33]. Binding was scored as negative when no rosettes were seen in the entire well. For erythrocyte-binding assay with monkey erythrocytes, macaque (*M. fascicularis*) erythrocytes (1% haematocrit in incomplete DMEM) were used. All erythrocyte-binding assays were performed in triplicates.

### Evaluation of the recombinant PkDBPαII binding specificity

To assess the binding specificity of the recombinant PkDBPαII, human and macaque erythrocytes were incubated with anti-Fy6 monoclonal antibody (1 mg/µl) at 1:2000 dilution in incomplete DMEM at 37 °C for 1 h prior to the incubation with the transfected COS-7 cells. Anti-Fy6, which recognizes the 2C3 epitope on the DARC N-terminal region, was kindly provided by Professor Laurent Rénia (Singapore Immunology Network, A*Star). Erythrocyte-binding assays using anti-Fy6-treated erythrocytes were performed as described above.

### Statistical analysis

Data were analysed using the SPSS (ver.20) statistical software (IBM Corp., Chicago, Illinois, USA) and Microsoft Excel 2016 (Microsoft, Redmond, WA, USA). For erythrocyte-binding assays, two-way ANOVA was used to compare the mean differences of each group. Differences of *P* < 0.05 were considered significant.

## Results

**Fig. 1:**
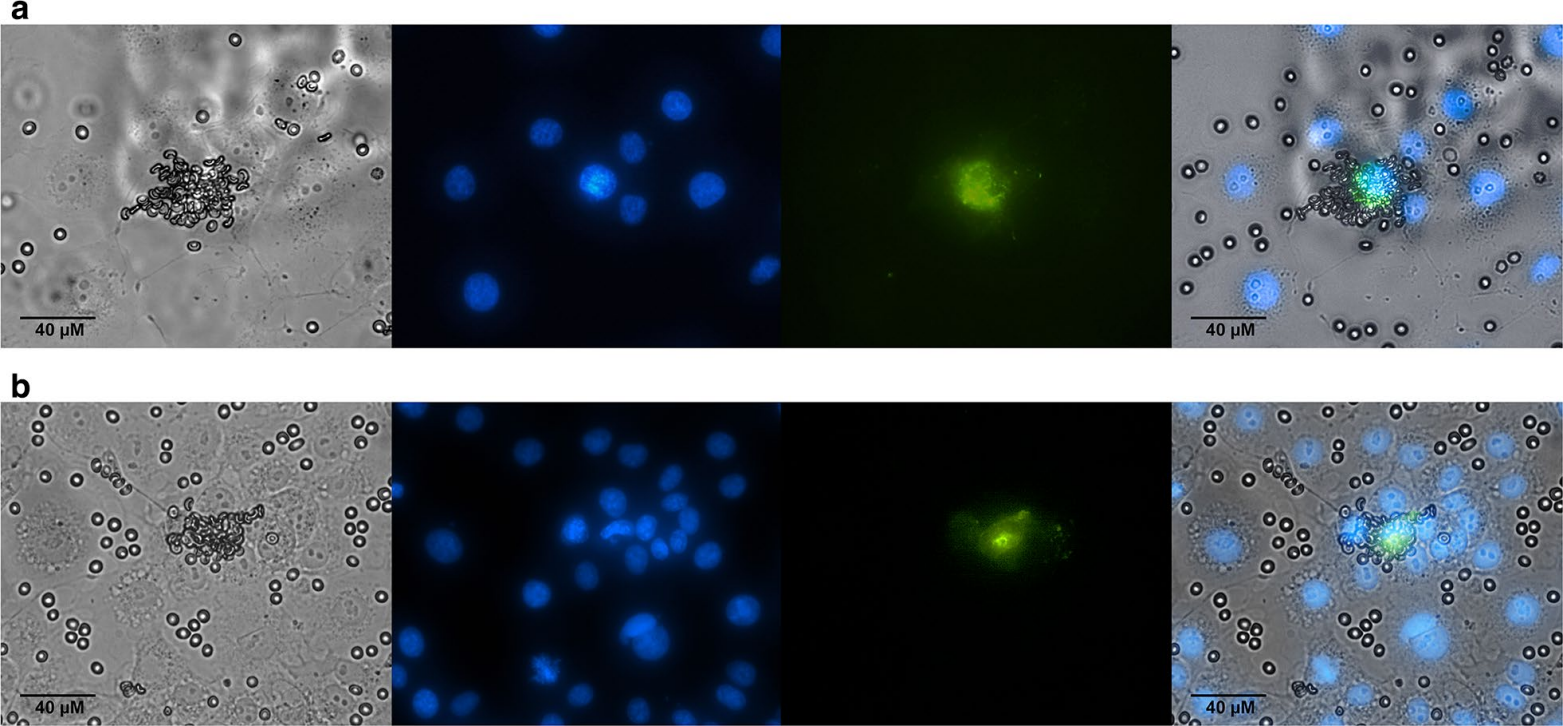
Binding of PkDBPαII expressed on transfected COS-7 cells with human Duffy-positive erythrocytes (a+b−) to form rosettes. **a** Transfected COS-7 cells expressing PkDBPαII of Peninsular Malaysia. **b** Transfected COS-7 cells expressing PkDBPαII from Malaysian Borneo. The transfected COS-7 cells (*green*) and the nuclei of COS-7 cells are visualized with Hoechst dye (*blue*). Images were captured using a Nikon ECLIPSE TE 300 inverted fluorescence microscope using the Plan Fluor ELWD 40×/0.45 aperture and ×10 magnification eye piece

The PkDBPαII of *P. knowlesi* isolates from Peninsular Malaysia and Malaysian Borneo were successfully transfected and expressed on COS-7 cells (Fig. 1). These transfected COS-7 cells were tested with erythrocytes in the erythrocyte-binding assays. In the negative controls which consisted of non-transfected COS-7 and COS-7 cells transfected with plasmid without inserts, did not produce any fluorescence.

**Fig. 2:**
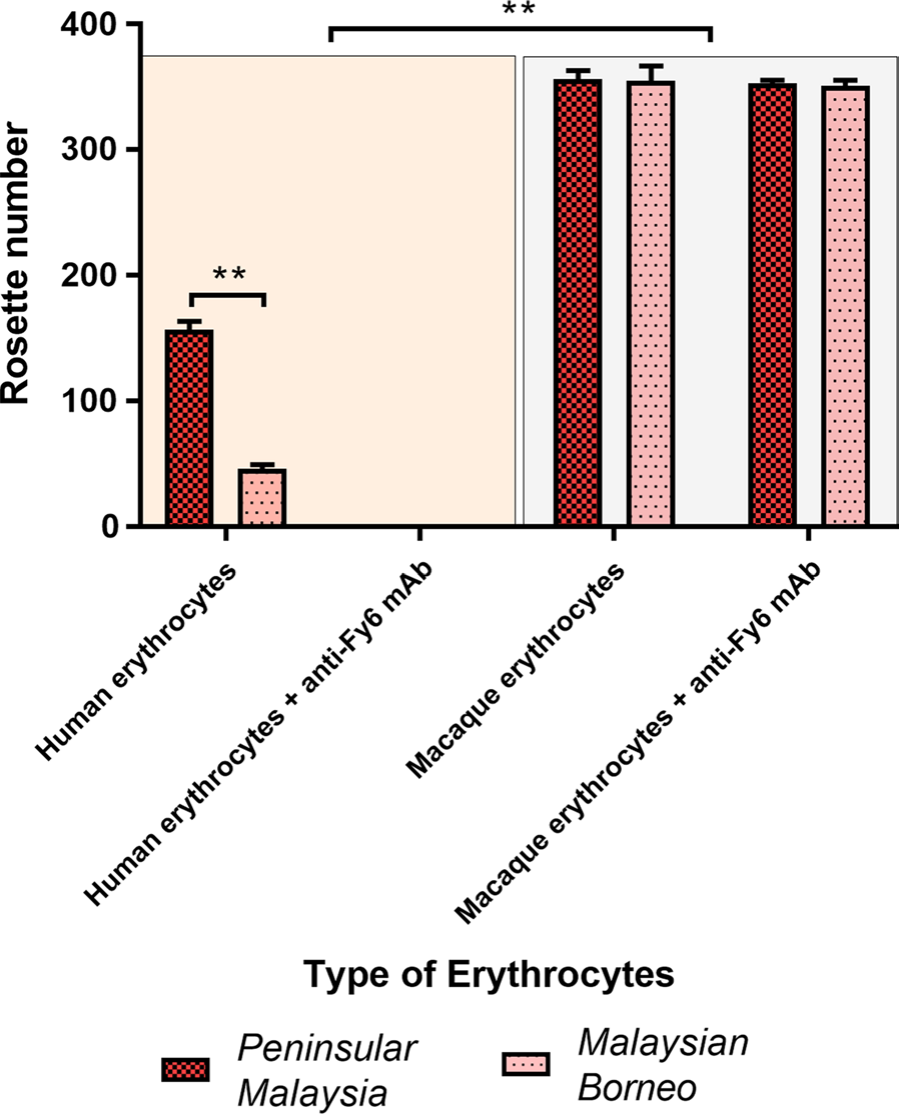
Binding activity level of PkDBPαII to human and macaque erythrocytes. Number of rosettes formed by COS-7 cells transfected with PkDBPαII of Peninsular Malaysia and Malaysian Borneo. A positive rosette was defined as more than half the surface of the transfected cells covered with attached erythrocytes, and the total number of transfected COS-7 cells counted for each independent experiment was 1500 per experiment. Data are shown as the mean rosettes number of three independent experiments. The *error bar* represents ±standard deviation. Statistical difference between PkDBPαII of Peninsular Malaysia and Malaysian Borneo, and between human and macaque erythrocytes is indicated with *double asterisk* (*P* < 0.001)

Recombinant PkDBPαII expressed on the COS-7 cell surface was able to bind to both human and macaque erythrocytes, but at different levels (Fig. 2). In the erythrocyte-binding assay using human erythrocytes, the binding activity or number of rosettes formed with PkDBPαII of Peninsular Malaysia was three times higher than that of Malaysian Borneo PkDBPαII (156.89 ± 6.62 rosettes and 46.00 ± 3.57 rosettes, respectively; *P* < 0.0001) (Table 1). When assayed with macaque erythrocytes, the PkDBPαII of Peninsular Malaysia (356.56 ± 6.75 rosettes) and Malaysian Borneo (355.22 ± 11.69 rosettes) showed similar binding activity levels (Table 1).

**Table 1:** Erythrocyte-binding assays of PkDBPαII of Peninsular Malaysia and Malaysian Borneo using various erythrocytes

Erythrocytes	Treatment	Haplotype	Binding^a^	Number of rosettes^b^
Human Fy(a+b−)	None	Peninsular	+	156.89 (±6.62)
	None	Borneo	+	46.00 (±3.57)
Macaque	None	Peninsular	+	356.56 (±6.75)
	None	Borneo	+	355.22 (±11.67)
Human Fy(a+b−)	Anti-Fy6	Peninsular	−	−
	Anti-Fy6	Borneo	−	−
Macaque	Anti-Fy6	Peninsular	+	352.67 (±2.89)
	Anti-Fy6	Borneo	+	351.83 (±4.36)

^a^ Binding of erythrocytes to COS-7 cells expressing PkDBPαII of Peninsular Malaysia and Malaysian Borneo; (+) Binding; (−) no binding

^b^ Number of COS7 cells with rosettes (mean ± SD) seen in 35–40 fields at 200× magnification by fluorescence microscopy. (+) A positive rosette was defined as more than half the surface of the transfected cells covered with attached erythrocytes, (−) No rosettes seen in the entire well

**Fig. 3:**
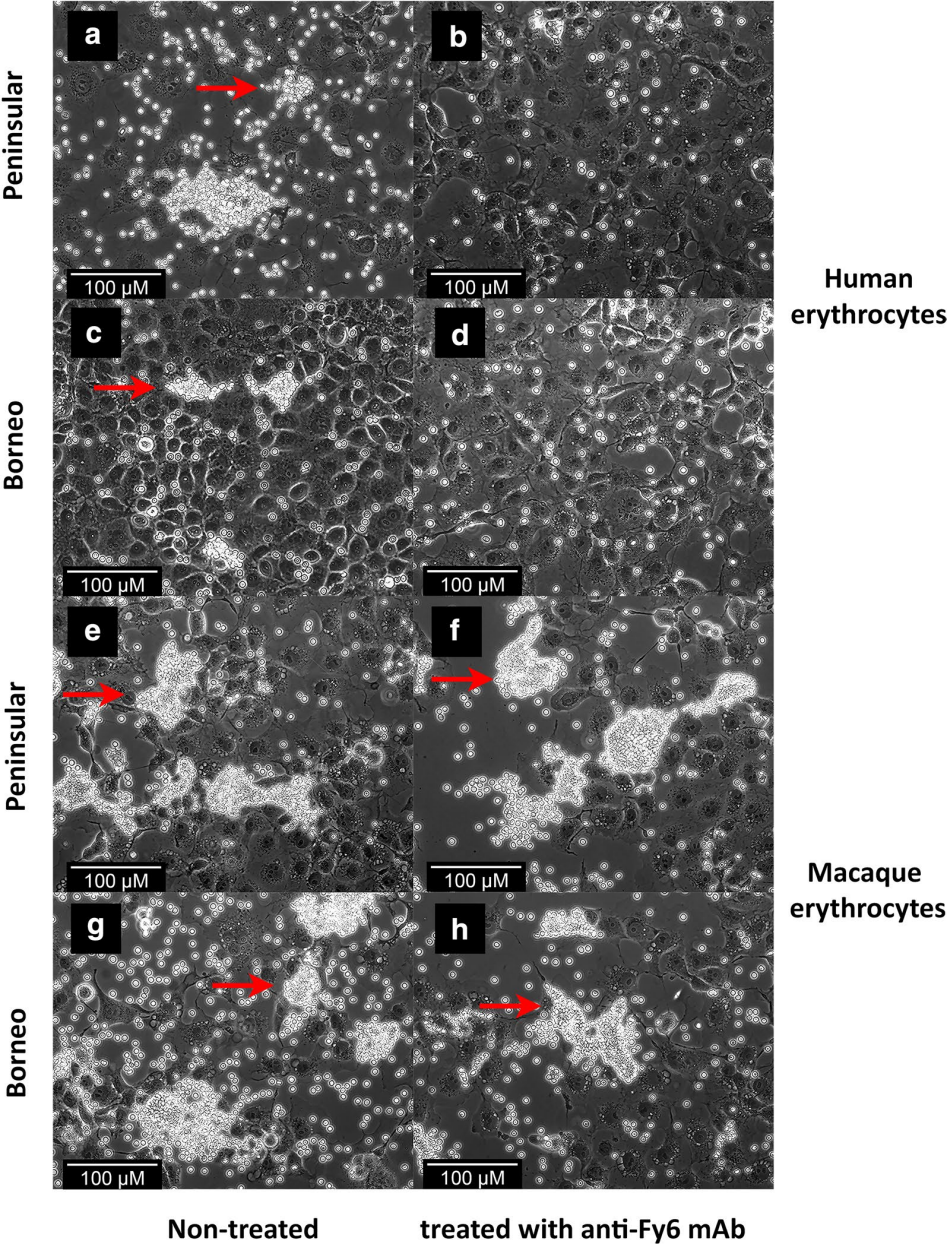
Binding inhibition of PkDBPαII to erythrocytes by anti-Fy6 treatment. **a**–**d** PkDBPαII incubated with 1% human erythrocytes; **e**–**h** PkDBPαII incubated with 1% macaque erythrocytes; **a**, **b**, **e**, **f** COS-7 cells transfected with PkDBPαII of Peninsular Malaysia; **c**, **d**, **g**, **h** COS-7 cells transfected with PkDBPαII of Malaysian Borneo; **a**, **c**, **e**, **g** erythrocytes not treated with anti-Fy6; **b**, **d**, **f**, **h** erythrocytes treated with anti-Fy6. Rosettes (*red arrows*) observed under inverted light microscope at ×200 magnification. No rosettes seen in **b** and **d**. Rosettes are more saturated on macaque erythrocytes

A change in binding activity was observed when the human erythrocytes were treated with anti-Fy6 prior to erythrocyte-binding assay. The binding activity with PkDBPαII of Peninsular Malaysia and Malaysian Borneo was completely eliminated (Fig. 3a–d). However, anti-Fy6 treatment of macaque erythrocytes had no effect on the binding activity of PkDBPαII (Fig. 3e–h). The PkDBPαII expressed on the surface of COS-7 cells bound to both non-treated and anti-Fy6-treated macaque erythrocytes regardless of the expressed PkDBPαII haplotype. In fact, the binding activity using macaque erythrocytes were significantly higher than those incubated with human erythrocytes (Table 1). Furthermore, the intensity of rosette formation in the PkDBPαII of Peninsular Malaysia and Malaysian Borneo was different when incubated with human and macaque erythrocytes. The rosettes formed on COS-7 cells with macaque erythrocytes were generally larger when compared to the rosettes formed with human erythrocytes irrespective of the PkDBPαII haplotypes (Fig. 3).

## Discussion

H2 and H47 are the predominant PkDBPαII haplotypes in Peninsular Malaysia and Malaysian Borneo, respectively. These haplotypes were chosen for this study, with the aim of determining whether haplotypic differences would affect PkDBPαII binding activity with erythrocytes.

**Fig. 4:**
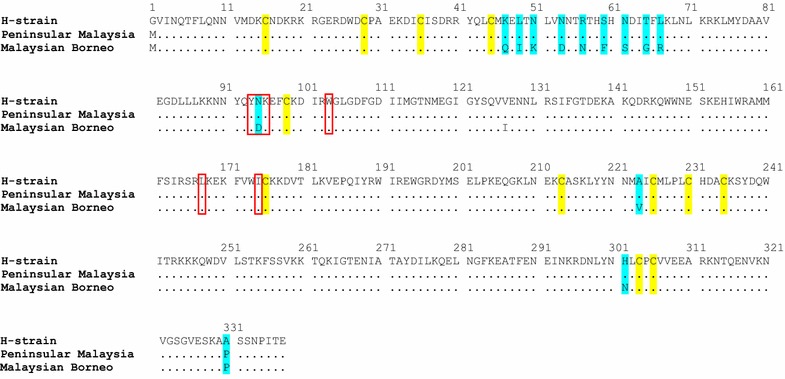
Sequence alignment of PkDBPαII from Peninsular Malaysia and Malaysian Borneo and the reference *P. knowlesi* strain H. Amino acid residues identical to those of the reference strain H (GenBank Accession No. M90466) are marked by *dots*. The amino acid changes and 12 highly conserved cysteine residues are highlighted in *cyan* and *yellow*, respectively. Amino acid residues, Y94, N95, K96, R103, L168 and I175, which have been identified previously as essential for binding with DARC [33, 34], are in *red boxes*

Results from this study revealed higher binding activity of PkDBPαII of Peninsular Malaysia with human Duffy-positive erythrocytes, as compared to Malaysian Borneo PkDBPαII. The PkDBPαII expressed on the COS-7 cell surface was based on the gene region defined from previous studies [30, 31]. This region encompasses a subdomain that provides the essential amino acid residues for proper folding and binding with the DARC on human erythrocytes. Within this subdomain, there are 12 cysteine residues (at positions 16, 29, 36, 45, 99, 176, 214, 226, 231, 235, 304, 306) that form 6 disulphide bridges which contribute to the tertiary structure of PkDBPαII for interaction with DARC. These cysteine residues have been shown to be conserved in the PkDBPαII of Peninsular Malaysia and Malaysian Borneo [28]. Amino acid residues Y94, N95, K96, R103, L168 and I175 in this subdomain have been identified as essential for binding with DARC (Fig. 4). Sequence alignment revealed that these residues were conserved except at position 95, where N was substituted with D in the PkDBPαII of Malaysian Borneo (Fig. 4). Apart from this N95D substitution, 11 other amino acid differences were identified between the PkDBPαII of Peninsular Malaysia and Malaysian Borneo (Fig. 4). These amino acid substitutions may have caused changes in the conformational structure of PkDBPαII of Malaysian Borneo, thus affecting its ability to bind to human erythrocytes efficiently.

Surprisingly, the binding activity level of PkDBPαII of Peninsular Malaysia and Malaysian Borneo to macaque erythrocytes was similar. The reason for this is unclear, but it may be plausible that amino acids for interaction with macaque erythrocyte receptor may be different from those of human erythrocyte. Therefore, the non-synonymous mutations in the PkDBPαII of either Peninsular Malaysia or Malaysian Borneo have no effect on the binding level of the protein to macaque erythrocytes.

The PkDBPαII plays a vital role in the invasion of parasites by mediating interaction with its corresponding receptor, the Duffy antigens on the surface of human erythrocytes. Previous studies have shown that human Duffy-negative Fy(a−b−) erythrocytes are refractory to invasion by *P. knowlesi* and *P. vivax* merozoites, while human Duffy-positive erythrocytes are susceptible [32, 33]. *Plasmodium knowlesi* merozoites require the interaction between PkDBPαII with Duffy determinant Fy^a^ or Fy^b^ for binding with erythrocytes. Thus, by blocking these determinants, it would inhibit the binding. This inhibition was observed in the present erythrocyte-binding assay using anti-Fy6 treatment. The Fy6 epitope is located at the N-terminal extracellular domain of DARC in close proximity to the Fy^a^/Fy^b^ determinant [34]. A previous study has shown that incubation with anti-Fy6 resulted in full inhibition of binding activity and invasion by *P. knowlesi* into human erythrocytes [35]. The inability of PkDBPαII of Peninsular Malaysia and Malaysian Borneo to bind to anti-Fy6 treated human erythrocytes demonstrated that the expressed PkDBPαII bound to the Duffy determinants of human DARC and not to other available receptors, such as sialic acid residues [36, 37].

In the present study, treatment of macaque erythrocyte with anti-Fy6 did not affect its binding with PkDBPαII. The Fy6 determinant is present on all human erythrocytes except those with Fy(a−b−) phenotype. In contrast, the Fy6 determinant is present only on the erythrocytes of some non-human primate species such as the Great Apes and New World monkeys. Erythrocytes of *M. fascicularis* are Fy^b^ but lack the Fy6 determinant [38]. Hence, the inability of anti-Fy6 to hinder interaction of PkDBPαII with *M. fascicularis* erythrocytes is due the absence of the Fy6 determinant. With the Fy^b^ determinant on erythrocytes still exposed, the binding of PkDBPαII with treated *M. fascicularis* erythrocytes occurred successfully.

The present study also demonstrated preferential binding activity of PkDBPαII. There was lesser number of rosettes in the erythrocyte-binding assay with human erythrocytes compared to macaque erythrocytes. This may suggest that the PkDBPαII binds to different Duffy determinants on human and macaque erythrocytes. This finding is in agreement with a previous study that postulated alternative determinants on DARC for binding [38].

The human Duffy-positive Fy(a+b−) erythrocytes were chosen in the present study primarily because this phenotype is the most common in the Malaysian population. Nonetheless, it would be worthwhile in future studies to investigate other Duffy phenotypes such as Fy(a+b+), Fy(a−b+) and Fy(a−b−). In vitro invasion studies have shown reduced efficiency of *P. knowlesi* to invade human Fy(a+b−) compared with Fy(a−b+) erythrocytes [14]. Furthermore, Fy^a^ expressing erythrocytes show reduced binding to PvDBPII and reduced susceptibility to vivax malaria [39].

There has been postulation that enhanced virulence and multiplication rates of *Plasmodium parasites* may be results of genetic polymorphisms that improve binding ability of the parasites to human erythrocytes [40]. Clinical and epidemiological findings revealed high number of severe knowlesi malaria in Malaysian Borneo. Oddly, results from this study found lower binding activity level of the Malaysian Borneo PkDBPαII haplotype compared to that of Peninsular Malaysia. A study has reported that *P. knowlesi* normocyte binding protein (PkNBPXa) polymorphisms are important determinants of high parasitaemia and disease severity in *P. knowlesi* infection [41]. Recently, it has been demonstrated that PkNBPXa is required for human erythrocyte invasion, and suggested that its role occurs after initial merozoite attachment [40]. Nevertheless, further research needs to be conducted to determine whether there is interaction between PkDBPαII and PkNBPXa in the invasion process, and polymorphism of PkDBPαII in part contributes to disease severity.

## Conclusion

This study is the first report of phenotypic difference between PkDBPαII haplotypes. The biological implication of this finding is yet to be determined. Therefore, further studies need to be carried out to determine whether this differential binding level can be associated with severity of knowlesi malaria in human.

## Abbreviations

PkDBPαII: *Plasmodium knowlesi* Duffy binding protein α, region II
PvDBP: *Plasmodium vivax* Duffy binding protein
DARC: Duffy antigens receptors for chemokine
